# Molecular networks of the FOXP2 transcription factor in the brain

**DOI:** 10.15252/embr.202152803

**Published:** 2021-07-14

**Authors:** Joery den Hoed, Karthikeyan Devaraju, Simon E Fisher

**Affiliations:** ^1^ Language and Genetics Department Max Planck Institute for Psycholinguistics Nijmegen The Netherlands; ^2^ International Max Planck Research School for Language Sciences Max Planck Institute for Psycholinguistics Nijmegen The Netherlands; ^3^ Donders Institute for Brain, Cognition and Behaviour Radboud University Nijmegen The Netherlands

**Keywords:** FOXP2, molecular network, neurodevelopment, speech disorder, transcription factor, Chromatin, Epigenetics, Genomics & Functional Genomics, Neuroscience

## Abstract

The discovery of the FOXP2 transcription factor, and its implication in a rare severe human speech and language disorder, has led to two decades of empirical studies focused on uncovering its roles in the brain using a range of *in vitro* and *in vivo* methods. Here, we discuss what we have learned about the regulation of *FOXP2*, its downstream effectors, and its modes of action as a transcription factor in brain development and function, providing an integrated overview of what is currently known about the critical molecular networks.

## Graphical abstract

FOXP2 was discovered 20 years ago to be required for human speech and language development. This review discusses molecular networks of FOXP2 regulation, downstream effectors, and how it acts as a transcription factor in brain development and function.

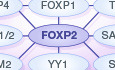

**Glossary**
ADHD: attention‐deficit/hyperactivity disorder
BCL11B: B‐cell lymphoma/leukemia 11B
BRET: bioluminescence resonance energy transfer
CASK: calcium/calmodulin‐dependent serine protein kinase 3
CHD: chromodomain‐helicase‐DNA‐binding protein
ChIP: chromatin immunoprecipitation
CNTNAP2/CASPR2: contactin‐associated protein‐like 2
CTBP: C‐terminal‐binding protein
DISC1: disrupted in schizophrenia 1
FOXP: forkhead box/winged‐helix protein
GATAD2B: GATA zinc finger domain‐containing 2B
GRIN2A: glutamate ionotropic receptor NMDA type subunit 2A
GSK3β: glycogen‐synthase kinase 3 beta
HDAC: histone deacetylase
Int.: protein interactors
LZ: leucine zipper
MET: MET proto‐oncogene, receptor tyrosine kinase
MRI: magnetic resonance imaging
MTA: metastasis‐associated protein
NEDD9: neural precursor cell expressed developmentally downregulated protein 9
NEUROD: neurogenic differentiation 1
NFI: nuclear factor 1
NGN2: neurogenin 2
NR2F: nuclear receptor subfamily 2, group F
NuRD: nucleosome remodeling and histone deacetylase
PAX6: paired box protein 6
pcw: post‐conception week
PIAS: protein inhibitor of activated STAT
POU3F2: POU class 3 homeobox 2
PTM: post‐translational modification
RAR: retinoic acid receptor
RELN: reelin
ROR: RAR‐related orphan receptor
SATB: special AT‐rich binding protein
SNP: single nucleotide polymorphism
SOX5: SRY (sex determining region Y)‐box 5
SRPX2: sushi repeat‐containing protein X‐linked 2
SUMO: small ubiquitin‐like modifier
TBR: T‐box, brain
TCF/LEF: T‐cell factor/lymphoid enhancer‐binding factor
TF: transcription factor
VLDLR: very‐low‐density lipoprotein receptor
WNT: wingless‐related MMTV integration site 1
WNT3: wnt family member 3
YY1: yin yang 1
ZBTB20: zinc finger and BTB domain‐containing 20
ZF: zinc finger
ZMYM2: zinc finger MYM‐type protein 2

## Introduction

*FOXP2* was the first gene to be clearly linked to speech and language development. The initial finding was made through studies of a large multi‐generational family (the KE family) with a severe dominantly inherited developmental speech and language disorder (MIM #602081) (Lai *et al*, 2001). All fifteen affected family members carried a heterozygous missense mutation (p.R553H) disrupting *FOXP2*. In the two decades since then, additional cases of FOXP2‐related speech and language disorders have been discovered, both inherited and *de novo* (MacDermot *et al*, 2005; Feuk *et al*, 2006; Reuter *et al*, 2017), with childhood apraxia of speech (also called developmental verbal dyspraxia) as a core phenotypic feature, characterized by difficulties in coordinating sequences of articulatory movements underlying proficient speech. In a subset of individuals, broader phenotypes are observed including oral motor deficits, global developmental delays, and/or autism spectrum disorder (Morgan *et al*, 2016). Beyond the well‐documented consequences of rare highly penetrant genetic disruptions, studies have investigated contributions of common variation in *FOXP2* to genetically complex traits. For example, some studies of small samples proposed that single nucleotide polymorphisms (SNPs) in the *FOXP2* gene are associated with schizophrenia risk (Spaniel *et al*, 2011; Li *et al*, 2013; Rao *et al*, 2017), but there is little evidence of replication (Yin *et al*, 2018). Large‐scale systematic genome‐wide association studies have identified significant associations of intronic *FOXP2* SNPs with several traits, including attention‐deficit/hyperactivity disorder (ADHD) (Demontis *et al*, 2019) and risk‐taking behaviors (Clifton *et al*, 2018). Although rare disruptions in *FOXP2* have been associated with changes in brain activity (Liégeois *et al*, 2003) and structure (Watkins *et al*, 2002; Liégeois *et al*, 2016; Argyropoulos *et al*, 2019), common variation could not be linked to task‐based neural activations on language tasks (Uddén *et al*, 2019) or neuroanatomical differences between individuals (Hoogman *et al*, 2014).

FOXP2 belongs to the forkhead box/winged‐helix (FOX) family of proteins, a large group of transcription factors that share a highly conserved DNA‐binding domain of ˜ 80–100 amino acids, called the forkhead box (Weigel & Jackle, 1990; Hannenhalli & Kaestner, 2009) (following nomenclature guidelines, we use FOXP2 for humans, Foxp2 for mice, and FoxP2 for other species). There are 19 subclasses of FOX proteins, from FOXA to FOXS (Kaestner *et al*, 2000; Hannenhalli & Kaestner, 2009), with important roles in various biological processes, including cell differentiation, proliferation, and development (Hannenhalli & Kaestner, 2009; Zhang *et al*, 2017). Although they all share a characteristic DNA‐binding domain, different FOX proteins have distinct expression patterns and are involved in diverse mechanisms (Benayoun *et al*, 2011).

**Figure 1:**
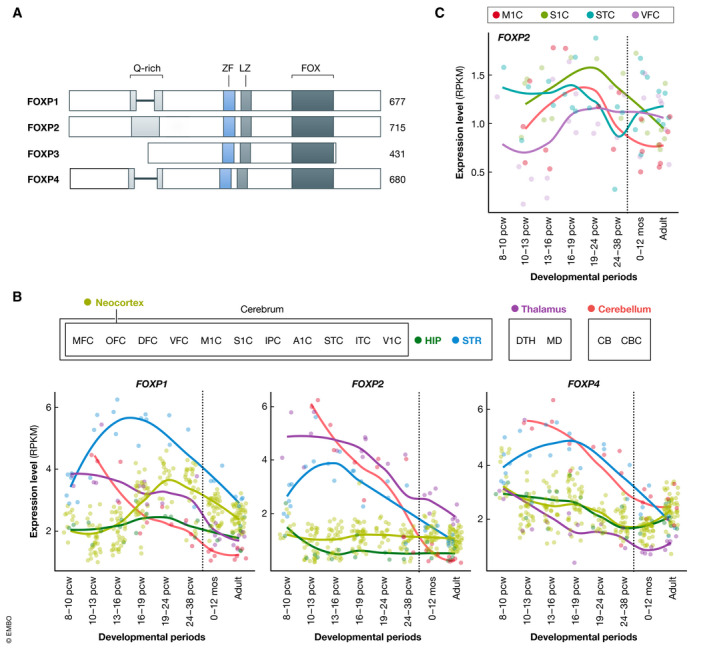
FOXP expression in the brain (A) Schematic representation of the FOXP family of proteins. The polyglutamine‐rich region is shaded in light gray (Q‐rich), the zinc finger domain in light blue (ZF), the leucine zipper in regular gray (LZ), and the forkhead domain in dark gray (FOX). (B) Expression patterns of *FOXP1*, *FOXP2,* and *FOXP4* in the brain, based on the developmental human RNA sequencing dataset of BrainSpan (http://www.brainspan.org/). (C) Expression patterns of *FOXP2* in a selection of cortical regions. These regions were selected based on structural MRI studies with KE family members carrying a *FOXP2* mutation (Vargha‐Khadem *et al*, 1998; Watkins *et al*, 2002; Belton *et al*, 2003): Gray matter differences were found in the cortical motor‐related areas, the inferior frontal gyrus and the superior temporal gyrus, among other regions. While the expression in the primary motor cortex (M1C) and the primary sensory cortex (S1C) peaks during development, the expression of *FOXP2* in the superior temporal cortex (STC) and the ventromedial prefrontal cortex (VFC) seems to be maintained during adulthood. (B, C) Each individual dot represents a brain sample, and the lines are loess curves fitted through the data points. The dashed vertical line represents time of birth. Abbreviations for the analyzed brain regions are A1C, primary auditory cortex; CB, cerebellum; CBC, cerebellar cortex; DFC, dorsolateral prefrontal cortex; DTH, dorsal thalamic nucleus; HIP, hippocampus; IPC, inferior parietal cortex; ITC, inferior temporal cortex; M1C, primary motor cortex; MD, mediodorsal thalamic nucleus; MFC, medial frontal cortex; OFC, orbitofrontal; S1C, primary sensory cortex; STR, striatum; TC, superior temporal cortex; V1C, primary visual cortex; VFC, ventromedial prefrontal cortex. Other abbreviations are mos, months; pcw, post‐conception week.

The FOXP subclass comprises four members, FOXP1–4 (Shu *et al*, 2001; Li *et al*, 2004). As well as the DNA‐binding domain, FOXP proteins share a zinc finger and leucine zipper motif (Fig 1A) (Wang *et al*, 2003; Li *et al*, 2004). Moreover, FOXP1, FOXP2, and FOXP4 contain long N‐terminal glutamine‐rich regions of unknown function (Wang *et al*, 2003; Li *et al*, 2004). A unique feature of the FOXP subclass is that they form homo‐ and heterodimers via the conserved leucine zipper, which appears essential for DNA binding and transcription regulation (Li *et al*, 2004). They may even form oligomer complexes, as detected for FoxP1, FoxP2, and FoxP4 in studies of zebra finch brain (Mendoza & Scharff, 2017). Formation of FOXP homo‐ and heterodimers in any particular tissue/cell type is likely mediated by expression and availability of the different FOXP proteins, providing potential for more complex regulation of downstream pathways.

While FOXP3 expression and function is largely limited to the immune system (Fontenot *et al*, 2003), FOXP1, FOXP2, and FOXP4 are expressed in various tissues throughout the body, including the brain, where they show distinctive, yet partially overlapping, expression patterns (human fetal and post‐natal expression of *FOXP1*, *FOXP2,* and *FOXP4* based on BrainSpan expression data: Fig 1B and C. For a detailed review on the expression patterns of *FOXP* genes in the brain, see (Co *et al*, 2020)). FOXP1 expression is enriched in layers III‐IV of the cerebral cortex (Ferland *et al*, 2003; Hisaoka *et al*, 2010), as well as the thalamus, striatum, and CA1 subregion of the hippocampus (Ferland *et al*, 2003). Main sites of FOXP2 expression include layers IV‐VI of the cerebral cortex (Ferland *et al*, 2003; Lai *et al*, 2003; Campbell *et al*, 2009; Hisaoka *et al*, 2010), the striatum (Ferland *et al*, 2003; Lai *et al*, 2003; Campbell *et al*, 2009; Garcia‐Calero *et al*, 2016), the posterior and lateral thalamic nuclei (Ferland *et al*, 2003; Lai *et al*, 2003; Campbell *et al*, 2009), the Purkinje cells in the cerebellum (Lai *et al*, 2003; Campbell *et al*, 2009), and the inferior olive (Ferland *et al*, 2003; Lai *et al*, 2003; Campbell *et al*, 2009). FOXP4 has been less well studied than the other FOXP proteins, but is expressed in the subventricular zone, throughout the cortical plate and in the striatum during embryonic development (Takahashi *et al*, 2008), and in Purkinje cells (Tam *et al*, 2011).

The roles of *FOXP2* have been investigated by studying its orthologues in an array of animal models. Mice that lack both alleles of *Foxp2* have severe motor impairments, developmental delays, and typically die by post‐natal day 21 (Shu *et al*, 2005), while heterozygous animals show no obvious differences compared to wild‐type littermates, but display some altered vocal behaviors (Castellucci *et al*, 2016). Mice that are heterozygous for the mutation originally identified in the KE family display reduced motor‐skill learning (Groszer *et al*, 2008) and produce shorter sequences of ultrasonic vocalizations with less complex syntax (Chabout *et al*, 2016), as compared to wild‐type littermates. *Foxp2* expression in the mouse cortex, striatum, and cerebellum modulates different aspects of motor function, as demonstrated by conditional homozygous knockouts targeting these structures (French *et al*, 2019). However, selective deletion of the gene in each of these brain regions does not significantly alter production of ultrasonic vocalizations (Urbanus *et al*, 2020). Interestingly, while selective deletion of *Foxp2* in the mouse cortex does not appear to impact development of cortical structures during embryogenesis (Co *et al*, 2019; Kast *et al*, 2019), cortical‐specific knockouts are reported to nonetheless show altered social behaviors (Co *et al*, 2019; Medvedeva *et al*, 2019). When mouse *Foxp2* is constitutively replaced by a partially humanized version, medium spiny neurons in the striatum show increases in dendrite length and synaptic plasticity (Enard *et al*, 2009), consistent with multiple studies implicating the gene in development and function of corticostriatal circuitry (Vernes *et al*, 2011; French *et al*, 2012; Chen *et al*, 2016; Hachigian *et al*, 2017; van Rhijn *et al*, 2018; French *et al*, 2019). Moreover, knockdown and overexpression studies in the brains of zebra finches suggest that avian *FoxP2* is important not only in auditory‐guided vocal learning during development, but also for maintenance of vocal behaviors in adulthood (Haesler *et al*, 2004; Heston & White, 2015; Day *et al*, 2019; Norton *et al*, 2019; Xiao *et al*, 2021).

Notably, in humans, heterozygous disruptions of *FOXP1* and *FOXP4* have also been linked to neurodevelopmental disorders: an intellectual disability syndrome, frequently accompanied with autistic features and language impairment (MIM #613670) (Hamdan *et al*, 2010; O'Roak *et al*, 2011; Srivastava *et al*, 2014; Lozano *et al*, 2015; Sollis *et al*, 2016), and a milder developmental disorder with speech/language delays and congenital abnormalities (Snijders Blok *et al*, 2021), respectively. Some of the etiological variants affect equivalent residues in the DNA‐binding domain of these genes (Sollis *et al*, 2017; Snijders Blok *et al*, 2021). While differences in the associated phenotypes are likely explained by the distinct expression patterns of the FOXP proteins, there are also regions of overlap where they can potentially form heterodimers. More thorough phenotypic comparison studies between these distinct neurodevelopmental disorders and functional follow‐up would be required to uncover whether equivalent variants in *FOXP1* and *FOXP4* directly impact speech and language or whether they have an indirect effect on the function of FOXP2.

In‐depth studies of the functions of FOXP2 and its orthologues in brain development have involved not only mice and zebra finches (as noted above), but also other models such as zebrafish and cell‐based systems. These investigations have uncovered upstream regulators of its expression, downstream targets that it regulates, and protein interactions that modulate its functions. Here, we give an up‐to‐date overview of the molecular networks of FOXP2 in the brain, highlighting how this information promises to deliver novel insights into roles of the gene in cognition and behavior.

## Regulation of *FOXP2* expression

Although the specific spatiotemporal expression patterns of FOXP2 in the brain imply tight regulation, little is known about the upstream mechanisms involved. Only a few transcription factors have been shown to bind to the genomic locus and/or to directly regulate its expression.

### Tbr1 activates Foxp2 expression in the developing cortex

**Figure 2:**
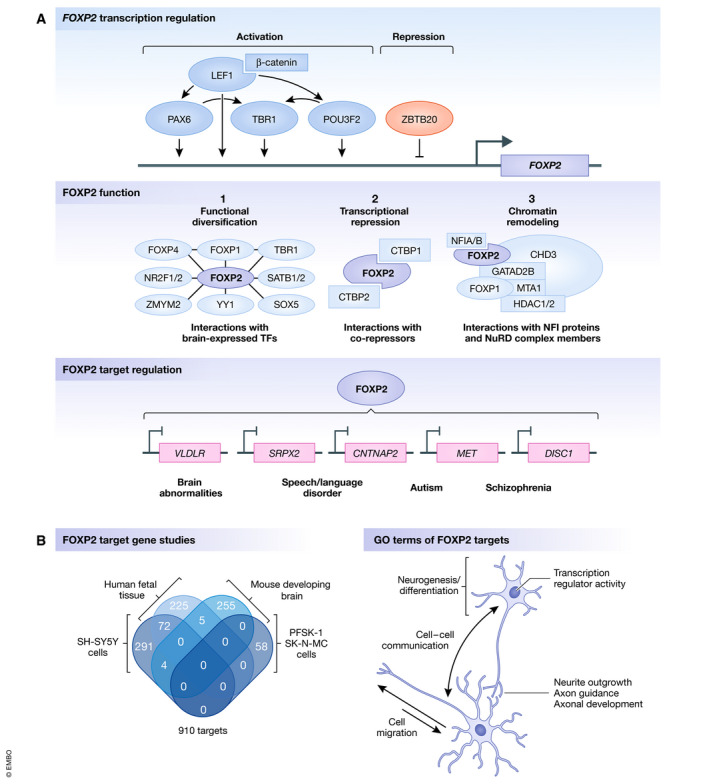
FOXP2 molecular networks (A) An overview of FOXP2 molecular networks in the brain, at the level of transcription regulation, function, and target regulation. This overview represents results from a selection of separate studies using different types of model systems. TFs: transcription factors. (B) Left, a Venn diagram showing the overlap between FOXP2 target genes identified in four FOXP2 ChIP‐chip/seq studies. SH‐SY5Y and SK‐N‐MC are human neuroblastoma cell lines, and PFSK‐1 is a neuroectodermal tumor cell line. Right, a schematic with a selection of gene ontology (GO) terms that are associated with the identified FOXP2 target genes.

TBR1 is a neural transcription factor with high expression in deep layers of the cortex, where it promotes a layer‐VI identity, largely via repression of layer‐V genes (Han *et al*, 2011; McKenna *et al*, 2011). In adult mice, almost 70% of FOXP2‐positive cells in layer VI express TBR1 (Medvedeva *et al*, 2019), and cell‐based assays have demonstrated that TBR1, in complex with its co‐regulator CASK, can activate *FOXP2* expression (Fig 2A) (Becker *et al*, 2018; Fazel Darbandi *et al*, 2018). Conditional deletion of *Tbr1* in layer‐VI neurons of mice leads to reduced *Foxp2* expression in these neurons, which shift to a layer‐V‐like identity (Fazel Darbandi *et al*, 2018). Although the role of *FOXP2* in cortical lamination is limited, based on studies with cortical‐specific knockout mice (Kast *et al*, 2019), the gene may be part of the regulatory program involved in formation, maintenance, and connectivity of corticothalamic neurons in layer VI (Druart *et al*, 2020), under control of TBR1. People with heterozygous *FOXP2* disruptions have been reported to show subtle differences in gray matter density in several parts of the cortex (Watkins *et al*, 2002), based on voxel‐based morphometry of MRI scans, although it is not known whether this involves altered connectivity and/or function of layer‐VI neurons in those regions. Recurrent *de novo* mutations of *TBR1* have been linked to a neurodevelopmental syndrome involving intellectual disability and/or autism spectrum disorder, and sometimes language impairments (MIM #606053), suggesting some phenotypic overlaps with FOXP2‐related disorder (Deriziotis *et al*, 2014).

### Regulation of FOXP2 by the canonical WNT/β‐catenin signaling pathway

The genomic region upstream of the *FOXP2* locus contains six highly conserved binding regions for TCF/LEF transcription factors (Hallikas *et al*, 2006; Bonkowsky *et al*, 2008), regulatory proteins that are activated by canonical WNT/β‐catenin signaling, and involved in proliferation and direction of cell fate (Bonkowsky *et al*, 2008). Binding of WNT to its receptor, Frizzled, leads to inhibition of GSK3β and accumulation of β‐catenin, which translocates to the nucleus and activates transcription via TCF/LEF transcription factors (Ciani & Salinas, 2005). One such TCF/LEF transcription factor is LEF1. *FoxP2* and *Lef1* are co‐expressed in the developing zebrafish brain, where knockdown of *Lef1* expression yields loss of *FoxP2* expression (Bonkowsky *et al*, 2008). Chromatin immunoprecipitation (ChIP) against Lef1 showed enrichment of the predicted Tcf/Lef binding regions upstream of *FoxP2*, suggesting that Lef1 directly binds to these enhancers to activate *FoxP2* expression (Bonkowsky *et al*, 2008).

The *FOXP2* locus also includes multiple highly conserved binding sites for PAX6, a key regulator of central nervous system development (Coutinho *et al*, 2011). Knockdown of *Pax6* in developing zebrafish embryos disrupts *FoxP2* expression, while for knockout mice lacking *Pax6*, expression of *Foxp2* is absent in the dorsolateral telencephalon (Coutinho *et al*, 2011). ChIP against *Pax6* in zebrafish embryos showed enrichment of binding sites in the *FoxP2* locus, confirming it as a direct target (Coutinho *et al*, 2011). In the developing neocortex, PAX6 is expressed in neural progenitor cells in the ventricular zone, regulating the cell cycle and differentiation (Gotz *et al*, 1998), while FOXP2 is expressed at low levels in progenitor cells (Tsui *et al*, 2013; Garcia‐Calero *et al*, 2016) but at higher levels in neurons in the cortical plate (Lai *et al*, 2003; Garcia‐Calero *et al*, 2016) and (as noted above) later in deep cortical layers (Hisaoka *et al*, 2010). Under control of WNT3, secreted by thalamic axons that grow into the developing neocortex, FOXP2 mRNA has been shown to be actively translated, driving differentiation of early neurons into deep layer neurons (Kraushar *et al*, 2015). Activation of FOXP2 by PAX6 might therefore be one of the steps that lead to differentiation of neural progenitor cells into neurons, fine‐tuning their activity and connectivity.

The middle of the *FOXP2* locus contains an intronic regulatory element with a binding site for POU3F2, a well‐known neural transcription factor (Maricic *et al*, 2013). This element drew the attention of molecular anthropologists studying the evolution of *FOXP2*, because the POU3F2‐binding site contains a DNA variant that arose specifically on the human lineage after splitting from our common ancestor with Neanderthals/Denisovans. However, the site is not fixed in modern human populations; analysis of next‐generation sequencing data from around the world shows that it remains polymorphic in southern Africa, casting doubt on the significance of this variant for human evolution (Atkinson *et al*, 2018) (see (Fisher, 2019) for a recent account of how views of the relevance of *FOXP2* for human evolution have shifted with the availability of comprehensive genome‐wide sequencing datasets and enhanced methods for assessing signals of selection). Based on reporter gene assays with the intronic enhancer, it has been suggested that binding of POU3F2 to this site may lead to increased FOXP2 expression (Maricic *et al*, 2013), although this finding has not been confirmed in a more physiologically relevant model and it is possible that the element instead regulates the expression of a different gene in the vicinity. Pou3f2 plays important roles in the formation and radial migration of upper‐layer cortical neurons (McEvilly *et al*, 2002; Sugitani *et al*, 2002) and is known to drive expression of *Ngn2*, *Tbr2,* and *Tbr1*, facilitating the differentiation of glutamatergic neurons (Dominguez *et al*, 2013).

PAX6 and POU3F2 are, like FOXP2, direct downstream targets of LEF1 (Goodall *et al*, 2004; Gan *et al*, 2014; Belinson *et al*, 2016). The LEF1‐β‐catenin/PAX6 signaling pathway is involved in self‐renewal of neural progenitors and neurogenesis during neocortical development, initiating the PAX6/NGN2/TBR2/NEUROD/TBR1 cascade (Gan *et al*, 2014). LEF1‐β‐catenin/POU3F2 signaling has been found to contribute to expansion of cortical neural progenitors and neurogenesis via the POU3F2/TBR2 and POU3F2/TBR1 cascades (Dominguez *et al*, 2013; Belinson *et al*, 2016). We speculate that FOXP2 and its transcriptional regulators LEF1, PAX6, and POU3F2 may all be downstream effectors of WNT/β‐catenin signaling (Fig 2A), a suggestion that could be tested in future with targeted experiments. Intriguingly, ectopic activation of Wnt signaling in the chicken optic cup has been shown to lead to upregulation of *FoxP2* expression (Trimarchi *et al*, 2009).

FOXP2 has been reported to regulate the transcription of WNT pathway genes and to directly interact with β‐catenin (Richter *et al*, 2021). Moreover, the FOXP2‐regulator TBR1 promotes maturation of layer‐VI cortical neurons by enhancing WNT signaling (Fazel Darbandi *et al*, 2020). As both an upstream and downstream player of this pathway, FOXP2 may potentially fulfill a central role in WNT/β‐catenin signaling in the brain, a hypothesis that would be interesting to explore with future studies.

### Zbtb20 represses Foxp2 expression in the hippocampus

To our knowledge, the only well‐characterized repressor of *FOXP2* identified through animal models is ZBTB20 (Fig 2A) (Nielsen *et al*, 2014), a transcription factor expressed in hippocampal projection neurons, cerebellar granular cells, and gliogenic progenitors (Mitchelmore *et al*, 2002). Zbtb20 was found to bind to and repress cortical layer marker genes, including *Foxp2*, in the developing mouse hippocampus, thereby directing a hippocampal fate while repressing other neuronal identities (Nielsen *et al*, 2014). Consistently, transgenic expression of *Zbtb20* in mice results in reduced *Foxp2* expression (Nielsen *et al*, 2014). Mouse Zbtb20 and human ZBTB20 proteins are highly conserved, with similar neural expression patterns (Nielsen *et al*, 2014), suggesting that the human orthologue may be important for *FOXP2* repression in the human hippocampus.

## Downstream effectors of FOXP2

Multiple studies have sought downstream neural targets of FOXP2, yielding insights into pathways that it regulates in the context of brain development, function, and disease.

### FOXP2 targets are important for neurite outgrowth and cell migration

In early work on identifying targets of FOXP2, three studies performed ChIP‐chip experiments on human fetal tissue (Spiteri *et al*, 2007), human neuroblastoma cells (Vernes *et al*, 2007), and embryonic mouse brain tissue (Vernes *et al*, 2011). Although no identified targets were common to all three studies, they are enriched for genes associated with similar gene ontology categories, namely cell communication/migration and nervous system development including neurogenesis, neurite development and axon guidance (Spiteri *et al*, 2007; Vernes *et al*, 2007; Vernes *et al*, 2011) (Fig 2B). A ChIP‐sequencing study of FOXP2 in neuroectodermal tumor cells and neuroblastoma cells identified 58 targets near high‐confidence ChIP peaks from a merged dataset, that were mostly enriched for genes linked to transcriptional (regulatory) activity (Nelson *et al*, 2013).

Follow‐up experiments confirmed that *Foxp2* promotes neurite outgrowth in both mouse neuroblastoma cells and mouse striatal primary neurons (Vernes *et al*, 2011). Indeed, genetic manipulations of *Foxp2* in an array of mouse models have been found to have effects on dendrite length. Specifically, introducing a partially humanized version of *Foxp2* into mice results in increased dendrite length of medium spiny neurons (Enard *et al*, 2009), while a loss‐of‐function mutation of the gene is reported to lead to decreased dendrite length of layer‐VI excitatory neurons in the cortex (Druart *et al*, 2020). The roles of *Foxp2* in neuronal migration are less clear‐cut; although *in vitro* studies support effects of the gene on cell migration phenotypes (Devanna *et al*, 2014), *in vivo* data from different mouse models are somewhat inconsistent with each other. For example, studies in which *Foxp2* expression was knocked down during embryonic development identified changes in cortical neurogenesis (Tsui *et al*, 2013) and in migration of neural progenitors out of the subventricular zone (Garcia‐Calero *et al*, 2016), but selective deletion of the gene was not found to have such effects (Kast *et al*, 2019).

Although large ChIP‐chip/sequencing datasets do not provide detailed directional insights into regulatory mechanisms, these data are valuable for further targeted investigations of relevant molecular pathways. In one such study, multiple targets from prior ChIP‐chip studies (Spiteri *et al*, 2007; Vernes *et al*, 2007; Vernes *et al*, 2011) were found to be differentially expressed in human neuroblastoma cells stably transfected with FOXP2, including retinoic acid signaling genes, such as the retinoic acid receptor (RAR)‐β, RAR‐related orphan receptor (ROR)‐α, ROR‐β, ROR‐γ, and NEDD9 (Devanna *et al*, 2014). Retinoic acid signaling is involved in forebrain and hindbrain development and directs the differentiation of embryonic stem cells into neural progenitors (Rhinn & Dolle, 2012). Retinoic acid treatment of human neuroblastoma cells induces neurite outgrowth and reduces cell migration, effects that are enhanced by concurrent FOXP2 overexpression (Devanna *et al*, 2014), suggesting that the gene may modulate retinoic acid signaling in the developing brain.

### FOXP2 target genes are implicated in neurodevelopmental disorders

Out of the hundreds of putative targets of FOXP2, a small subset have received special attention through validation and follow‐up in animal or cell‐based models. One of the first targets to be studied in this way was *CNTNAP2*, which encodes CASPR2, a neurexin transmembrane protein expressed widely in the brain, with roles in nerve conduction, neuronal migration, neurite outgrowth, and connectivity (Rodenas‐Cuadrado *et al*, 2014). FOXP2 directly binds to regulatory regions of the *CNTNAP2* locus to repress expression (Vernes *et al*, 2008; Mendoza & Scharff, 2017). This is consistent with complementary expression patterns reported for the two genes in human fetal cerebral cortex (Vernes *et al*, 2008) and increased *Cntnap2* expression in the cerebellum of a Foxp2‐R552H mouse model (based on the human KE family mutation) (Fujita *et al*, 2012). However, *CNTNAP2* expression changes temporally (Gordon *et al*, 2016) and expression patterns of these genes could potentially show different relationships at distinct stages of development and/or in different brain regions. Interestingly, a cluster of SNPs in *CNTNAP2* has been associated with reduced performance on a nonsense‐word repetition task in a cohort of children with developmental language disorders (Vernes *et al*, 2008) and with a measure of early communicative behavior in a general population sample (Whitehouse *et al*, 2011). Furthermore, homozygous and compound heterozygous loss‐of‐function variants cause a severe neurodevelopmental disorder with epilepsy and intellectual disability (MIM #610042) (Strauss *et al*, 2006; Zweier *et al*, 2009; Smogavec *et al*, 2016). Although in prior work both common and rare *CNTNAP2* variation has been linked to a range of brain‐related phenotypes (Fig 2A), including autism (MIM #612100) (Alarcon *et al*, 2008; Arking *et al*, 2008) and schizophrenia (Friedman *et al*, 2008; Ji *et al*, 2013), data from a recent large‐scale study argue that the contributions of this gene to risk of these psychiatric disorders have been overstated (Toma *et al*, 2018).

Other genes that are repressed by FOXP2, and where links have been investigated in follow‐up studies, include *SRPX2* (Roll *et al*, 2010), *MET* (Mukamel *et al*, 2011), and *DISC1* (Spiteri *et al*, 2007; Walker *et al*, 2012; Nelson *et al*, 2013). FOXP2 overexpression in cell‐based assays lowers the expression of *SRPX2* (Roll *et al*, 2010), *MET* (Mukamel *et al*, 2011), and *DISC1* (Walker *et al*, 2012), and FOXP2 directly binds to regulatory sequences in *MET* and *SRPX2* (Roll *et al*, 2010; Mukamel *et al*, 2011). Cell‐based assays additionally suggest that the FOXP2‐R553H mutation disrupts regulation of *SRPX2* and *DISC1* (Roll *et al*, 2010; Walker *et al*, 2012). *SRPX2* variants have been identified in people with epilepsy of the rolandic speech area, speech apraxia, polymicrogyria, and intellectual disability (MIM #300643) (Roll *et al*, 2006; Roll *et al*, 2010; Chen *et al*, 2017), although their etiological relevance is uncertain given subsequent discovery of *GRIN2A* disruptions in the affected individuals (Lesca *et al*, 2013). Common variation in *MET* has been associated with autism spectrum disorder (MIM %611015) (Campbell *et al*, 2006; Thanseem *et al*, 2010) and schizophrenia (Burdick *et al*, 2010), and post‐mortem brain studies have shown altered *MET* expression in individuals with autism (Campbell *et al*, 2007). The *DISC1* gene has been linked to schizophrenia (MIM #604906) (Hennah *et al*, 2003; Hodgkinson *et al*, 2004; Schumacher *et al*, 2009).

Beyond its effects as a transcriptional repressor, noted above, FOXP2 has been reported to be a direct activator of *VLDLR* expression (Spiteri *et al*, 2007; Vernes *et al*, 2007; Adam *et al*, 2016; Mendoza & Scharff, 2017). VLDLR is a receptor for RELN, expressed in the apical processes of migrating neurons in the developing cortex, with roles in neuronal migration, dendrite and spine development, and synaptic function (Lee & D'Arcangelo, 2016). Studies of zebra finch brain have found that FoxP2 protein directly binds to regulatory sequences of the *Vldlr* locus and that knockdown of the former reduces expression of the latter (Adam *et al*, 2016). Homozygous disruptions of the human *VLDLR* gene have been discovered in patients with cerebellar hypoplasia, mild cerebral gyral simplification, and intellectual disability (MIM #224050) (Boycott *et al*, 2005; Ozcelik *et al*, 2008; Dixon‐Salazar *et al*, 2012).

Based on data thus far collected on downstream pathways, FOXP2 and its targets belong to molecular networks that are crucial for aspects of brain function and that are implicated in a range of neurodevelopmental disorders with partially overlapping phenotypes, raising the possibility that etiological variants of these genes affect shared mechanisms (Fig 2A).

## FOXP2 transcriptional regulation

Although studies of FOXP2 have probed its expression patterns, regulation, and transcriptional targets, the molecular mechanisms by which this regulatory protein acts as a transcription factor have been much less explored.

### FOXP2 interacts with the CTBP transcriptional co‐repressors

The first proteins to be identified as putative binding partners of FOXP2 were CTBP1 and CTBP2 (Li *et al*, 2004), transcriptional co‐repressors that also interact with FOXP1 via a consensus binding site, which is lacking in FOXP4 (Li *et al*, 2004; Estruch *et al*, 2016a). *Drosophila* CtBP enhances repression by directly blocking the transcription initiation complex or inhibiting adjacent transcriptional activators (Nibu *et al*, 2003). Moreover, CTBP1 and CTBP2 were identified in a core protein complex that contained DNA‐binding proteins, histone‐modifying enzymes, histone methyltransferases, and chromodomain‐containing proteins (Shi *et al*, 2003), and may thereby aid FOXP2 in its transcriptional repressive functions (Fig 2A). Indeed, in cell‐based assays, CTBP1 is able to increase FOXP1 and FOXP2 repression of reporter constructs (Li *et al*, 2004). The FOXP2‐R553H protein, which harbors an etiological substitution disrupting the DNA‐binding domain (Vernes *et al*, 2006), retains its ability to bind to CTBP1 and CTBP2, suggesting that DNA binding of FOXP2 is not essential for the CTBP‐FOXP2 interaction (Estruch *et al*, 2016a). Since CTBP proteins depend on their interaction partners to be recruited to DNA, and FOXP2‐R553H is unable to bind to DNA, it is unlikely that this complex represses target genes.

### SUMOylation of FOXP2 modulates its function

Post‐translational modifications are another way to dynamically regulate transcription factor activity. One such modification is SUMOylation, the reversible coupling of small ubiquitin‐like modifiers (SUMOs), which are ubiquitously expressed polypeptides, to specific sites in proteins. FOXP2 has a SUMOylation site at position K674, which is SUMOylated by SUMO1/2/3 via interaction with PIAS1/3 (Estruch *et al*, 2016b; Usui *et al*, 2017). K674 SUMOylation is not critical for FOXP2 protein stability, dimerization, and subcellular localization in human cell lines (Estruch *et al*, 2016b; Meredith *et al*, 2016), but may alter its transcriptional activity (Meredith *et al*, 2016). Although one study did not detect changes in transcriptional repression of a non‐SUMOylated FOXP2 K674R mutant (Estruch *et al*, 2016b), another found this mutant to be less effective in repressing target promoters compared to wild‐type protein (Meredith *et al*, 2016). Disrupting the equivalent SUMOylation site in FOXP1 (K670) abolishes FOXP1 repression, while K670 SUMOylation in wild‐type FOXP1 enhances binding to the CTBP1 co‐repressor (Rocca *et al*, 2017). Studies of mice suggest that FOXP2 SUMOylation in the cerebellum is important for Purkinje cell development and motor functions (Usui *et al*, 2017). In cell‐based studies, ubiquitination, another form of post‐translational modification, has been found for an alternatively spliced short isoform of unknown significance (FOXP2.10+), while the canonical isoform was not ubiquitinated (Vernes *et al*, 2006). Whether other post‐translational modifications beyond SUMOylation and ubiquitination, such as phosphorylation and acetylation, significantly contribute to regulation of FOXP2 functions has yet to be elucidated.

### FOXP2 interacts with other brain‐expressed transcription factors

A mass spectrometry study to characterize the FOXP2 interactome identified multiple transcription factors binding to FOXP2 in HEK293 cells, including NR2F1, NR2F2, SATB1, SATB2, SOX5, YY1, and ZMYM2 (Estruch *et al*, 2018). Foxp2 is co‐expressed with Sox5, Satb1, Satb2, and Nr2f1 in a subset of neurons in the mouse cerebral cortex and with Nr2f2 in Purkinje cells (Estruch *et al*, 2018). The interactions were validated in cell lines using bioluminescence resonance energy transfer (BRET) assays (Estruch *et al*, 2018). Additionally, the cortical transcription factor TBR1 was identified as a putative FOXP2 interactor in a yeast‐two‐hybrid assay (Sakai *et al*, 2011) and confirmed with BRET (Deriziotis *et al*, 2014). The etiological FOXP2 p.R553H mutation disrupts the interactions with these brain‐expressed transcription factors (Deriziotis *et al*, 2014; Estruch *et al*, 2018). The functional importance of these interactions for *in vivo* brain development has not yet been studied, but may contribute to diversification of FOXP2 activity, guiding the protein to specific transcriptional complexes, changing its affinity for certain targets, and/or helping to recruit transcriptional co‐factors (Fig 2A).

### FOXP2 regulatory activity may be mediated via the NuRD chromatin remodeling complex

FOXP1, FOXP2, and FOXP4 all interact with the nucleosome remodeling and histone deacetylase (NuRD) complex (Chokas *et al*, 2010), a multiprotein complex that couples two independent chromatin‐regulatory functions, (i) ATP‐dependent histone remodeling and (ii) histone deacetylation (Tong *et al*, 1998; Xue *et al*, 1998). The complex, involved in both activation and repression of genes (Basta & Rauchman, 2015), is the most abundant form of deacetylase in mammals (Torchy *et al*, 2015) and is linked to fundamental biological processes, including cell cycle progression, genomic integrity (Lai & Wade, 2011), and differentiation of embryonic stem cells (Basta & Rauchman, 2015; Torchy *et al*, 2015). FOXP1 interacts with NuRD complex members HDAC1/2, GATAD2B, and MTA1 (Chokas *et al*, 2010), FOXP4 with HDAC1/2 and GATAD2B (Chokas *et al*, 2010), and FOXP2 with GATAD2B (Chokas *et al*, 2010) and CHD3 (Estruch *et al*, 2016b). For FOXP1 and FOXP4, these interactions further reduce target gene expression in cell‐based reporter assays, suggesting that these NuRD complex interaction partners act as co‐repressors. For the FOXP2‐GATAD2B interaction however, assays found no evidence of synergistic repression (Chokas *et al*, 2010).

Interestingly, the NuRD complex plays an important role in the developing brain, apparent from the links of multiple of the core NuRD complex members with neurodevelopmental disorders that are characterized by features that partly overlap with the FOXP2‐associated phenotypes. Mutations in the *CHD4* gene result in an intellectual disability syndrome that includes global developmental delay and in some cases macrocephaly (MIM #617159) (Sifrim *et al*, 2016; Weiss *et al*, 2016). A mutation in *CHD3* was first discovered in a child with childhood apraxia of speech (Eising *et al*, 2019), whereafter additional etiological variants were found in a number of patients that displayed intellectual disability, accompanied by speech/language problems and brain abnormalities including both macrocephaly and microcephaly (MIM #618205) (Snijders Blok *et al*, 2018). Furthermore, *GATAD2B* disruptions have been identified in patients with intellectual disability and limited speech (MIM #615074) (de Ligt *et al*, 2012; Willemsen *et al*, 2013; Shieh *et al*, 2020).

In addition to the direct interactions of FOXPs with NuRD complex members, there are multiple indirect links. FOXP2 and the HDAC1/2 proteins share at least three common interaction partners, the cortical transcription factors YY1 (Yang *et al*, 1996; Yao *et al*, 2001; Estruch *et al*, 2018), SATB1 (Yasui *et al*, 2002; Estruch *et al*, 2018), and SATB2 (Gyorgy *et al*, 2008; Estruch *et al*, 2018). In layer‐IV neurons of the cortex, Satb2 has been shown to assemble the NuRD complex upstream of *Bcl11b*, resulting in *Bcl11b* repression, via the Satb2‐Hdac1 interaction (Britanova *et al*, 2008). Repression of BCL11B in SATB2‐positive neurons is an essential mechanism in cortical lamination, resulting in upper‐layer neuron specification (Britanova *et al*, 2008). In humans, YY1 (MIM #617557), SATB1 (MIM # 619228 and #619229), and SATB2 (MIM #612313) are all implicated in neurodevelopmental disorders (Bengani *et al*, 2017; den Hoed *et al*, 2021; Gabriele *et al*, 2017). Notably, SATB2 mutations cause severe language impairments (Zarate & Fish, 2017). Furthermore, CTBP2, a direct FOXP2 interactor and co‐repressor (Estruch *et al*, 2016a), interacts with several NuRD complex members, namely HDAC2, MTA2, GATAD2B, and CHD4 (Zhao *et al*, 2014). Whether these FOXP2 interactors interact with FOXP2 and the NuRD complex simultaneously has not been studied.

Most FOXP‐NuRD complex interactions have only been characterized in cell lines or in the context of lung function (another tissue where FOXP proteins are expressed) (Chokas *et al*, 2010), and the importance of such interactions for brain development remains to be uncovered. The NuRD complex plays major roles in the proliferation, migration, and differentiation of neurons (Nitarska *et al*, 2016), and interactions with cortical transcription factors, such as SATB2, seem to recruit it to specific targets (Britanova *et al*, 2008). Hence, the FOXP proteins (as homo/heterodimers or together with other co‐factors) may guide the NuRD complex to the DNA, to repress or activate target sequences via chromatin remodeling (Fig 2A). FOXP2 mutations may disrupt this mechanism by abolishing either DNA binding or interaction with NuRD complex members, resulting in abnormal regulation of downstream targets. Mutations in NuRD complex members may result in similar transcriptional regulatory defects, contributing to partial overlaps in the neurodevelopmental phenotypes that are associated with FOXP2, GATAD2B, and SATB2 mutations.

In addition to potential chromatin remodeling functions via interactions with the NuRD complex, FOXP2 has been reported to mediate chromatin accessibility by interacting with transcriptional co‐factors NFIA and NFIB in neuronal cell‐based models (Hickey *et al*, 2019). Direct interactions of FOXP2 with DNA were found to yield repression of proliferation‐promoting genes, while FOXP2‐NFI complexes activated expression of genes driving neuronal differentiation via chromatin alterations (Hickey *et al*, 2019). Although FOXP2‐R553H in complex with NFIA was still able to open chromatin, it did not activate gene expression. Thus, these data suggest the existence of distinct FOXP2 regulatory modes that together mediate target gene expression.

## Future perspectives

**Figure 3:**
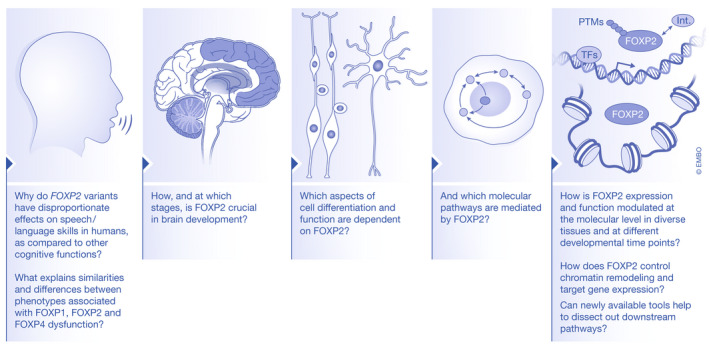
Open questions on the molecular aspects of FOXP2 in the brain Schematic with different levels of FOXP2 functioning. For each level, questions are included that have remained largely unanswered and should be focus of future studies. The shaded brain areas in the schematic in the second left panel represent regions of expression of FOXP2 that have been main focus in current literature. Int., protein interactors; PTMs, post‐translational modifications; TFs, transcription factors.

Two decades of molecular studies on the functions of FOXP2 have shown that it belongs to an extensive molecular network with brain‐expressed transcription factors and co‐regulators, mediating neuronal differentiation, neurite outgrowth and cell migration in human cell‐based assays, and shaping the development, plasticity and maturation of corticostriatal and corticocerebellar circuits important for behavioral phenotypes in animal models. Despite the attention FOXP2 has received over the years, much remains to be learned regarding its regulatory capabilities, position in molecular pathways, roles in cellular functions, and ultimately its effects on brain development and human speech and language capacities (Fig 3).

New and more sophisticated models may hold special promise for furthering our understanding of FOXP2 functions, particularly in light of links to speech and language. Human brain organoids grown from stem cells can model early stages of development of various parts of the nervous system (Kelava & Lancaster, 2016; Marton & Pașca, 2020) and overcome species‐specific developmental programs (Kanton *et al*, 2019), providing the opportunity to study the human transcriptome during brain development. Long‐term (Gordon *et al*, 2021) and slice cultures (Giandomenico *et al*, 2019; Qian *et al*, 2020) of these brain organoids result in maturation up to late fetal and early post‐natal stages, while merging of region‐specific organoids make it possible to model early establishment of brain circuitries (Andersen *et al*, 2020; Miura *et al*, 2020). Genetic manipulation of *FOXP2* in such model systems could reveal human‐specific functions that have been unable to be studied in traditional *in vitro* settings so far.

For studying FOXP2 functions *in vivo*, more relevant and non‐traditional animal models are also being explored (Lattenkamp & Vernes, 2018). In addition to zebra finches, other species of birds display auditory‐guided vocal learning (Pfenning *et al*, 2014), as well as bats (Knörnschild, 2014; Vernes, 2017) and ocean mammals (Ravignani *et al*, 2016). The latter two are evolutionarily closer to us, with brain structures and circuitries more similar to human brains. Indeed, analyses of FoxP expression patterns in the brains of bat species are already proving informative (Rodenas‐Cuadrado *et al*, 2018). Although the genetic tools in such species are not yet as well established as in the traditional animal models, optimization and validation of these in the coming years will open up exciting new avenues for investigations of FOXP2 and its orthologues, placing the critical molecular networks in their broader evolutionary context.

## Author contributions

All authors contributed to writing and revising of the manuscript and approved the final version.

## Conflict of interest

The authors declare that they have no conflict of interest.
